# Evaluation of a Rheumatology Transition Clinic

**DOI:** 10.1186/s12969-015-0016-x

**Published:** 2015-06-11

**Authors:** Elizabeth Stringer, Rachel Scott, Dianne Mosher, Inez MacNeill, Adam M Huber, Suzanne Ramsey, Bianca Lang

**Affiliations:** Division of Pediatric Rheumatology, IWK Health Centre, Halifax, NS B3K 6R8 Canada; Pediatric Endocrinology Service, CHU Sainte-Justine, 3175, chemin de la Cote-Sainte-Catherine, Montreal, QC H3T 1C5 Canada; Division of Rheumatology, University of Calgary, 3280 Hospital Drive NW, 3aa18 HRIC, T2N 4Z6 Calgary, AB Canada

**Keywords:** Adolescent rheumatology, Transition clinic, Transfer, Young adult

## Abstract

**Background:**

An adolescent with a chronic condition must prepare for transition from the pediatric to the adult health care system. Ideally, transition is a purposeful and coordinated process between the two systems. We sought to evaluate a pediatric rheumatology transition clinic from the perspective of the young adults who attended the clinic.

**Methods:**

Young adults who attended the IWK Health Centre Pediatric Rheumatology Transition Clinic in Halifax, Nova Scotia, Canada were asked to complete a mail questionnaire. In this clinic an adult rheumatologist joins the pediatric team for the patient’s visit. Subjects rated satisfaction with the clinic and how completely a number of items were addressed (e.g. knowledge about disease, self-management, adolescent issues) on a 10 cm visual analog scale (higher scores reflecting more favourable assessment). Compliance with follow-up post-transfer to adult care was assessed by self-report and a chart review. Data were summarized descriptively.

**Results:**

The response rate was 34 % (51/151). The mean age of respondents was 22 years with the majority diagnosed with juvenile idiopathic arthritis. Most patients were transferred to adult care between the ages of 17 and 20 years. The mean overall satisfaction score with the transition clinic was 7.3 ± 2.6. There was significant variability regarding how well individual transition-related items were perceived to have been addressed, with an overall mean of 6.1 ± 3.2. Items which received a majority of scores of > 7 included learning about side effects of medications, learning to live with their disease, confidence in disease management, and control of disease at transfer. Items rated as <5 by a third of respondents included addressing teen issues (smoking, alcohol, sexual health) and learning about new developments related to their condition. 74 % of patients reported regular appointments with adult rheumatology.

**Conclusions:**

Most young adults reported overall satisfaction with the transition clinic, however their perception of how adequately various transition issues were addressed was quite variable. It appears that there were some perceived deficits in the care that was provided in all areas, but possibly more so in counselling around general adolescent issues. There was a high rate of follow-up after transfer to the local adult clinic.

## Background

Adolescence is the period of transition from a dependent child to an independent adult. This normal developmental process includes changes in cognition from concrete to abstract thinking, moving towards increasing autonomy, identification of peer groups, as well as emotional, physical, and sexual development [1]. Chronic illness can affect an adolescent’s development in a number of ways including frequent and/or prolonged hospitalizations taking the youth out of school and away from peers, illness-related physical limitations, changes in physical appearance, and the natural tendency of families and health care providers to shelter a youth with a chronic disease [2]. In addition, some adolescents with a chronic illness may exhibit behaviors and attitudes that negatively impact a previously well-managed condition [3]. Taken altogether, adolescence can be a challenging period for youth with chronic illness, their families and their health care providers.

Adolescents with a chronic disease and their families must prepare for the transition from pediatric health care to the adult health care system. This process of transition is becoming increasingly recognized as an important area for clinicians, researchers, and policy-makers to address [4–9]. While acknowledging the need for more evidence, a number of position statements have been published outlining the fundamental principles of transition with one key message being that it should be a purposeful, organized, and coordinated process involving the youth, family and the health care team, with the goal of optimizing health and facilitating each young person’s attainment of their potential as they move into adulthood [4–9].

There is some evidence that morbidity increases in young adults once they transfer to adult care. Nakhla *et al.* found that diabetes-related hospitalizations increased in the 2 years after transfer compared with the 2 years before [10]. A study of children with renal transplants found that 35 % of grafts were lost following transfer to adult care with a median time to graft loss of 12 months, having had stable renal parameters for the 12 months before transfer [11]. The authors hypothesized that medication adherence played a significant role in graft loss. In a population of children with rheumatic diseases, active disease and disease flares were found to be high at the time of transfer [12]. Missed appointments can also be a problem in the transfer period [13].

In an effort to improve the transition process and ultimately the outcome of young adults with childhood-onset chronic diseases, specific clinics dedicated to facilitating the transition process have been developed in a number of centers. To date there has been minimal formal evaluation of these clinics. The aim of this study was to evaluate a pediatric rheumatology transition clinic. We modeled the transition clinic based on previously gathered information from adolescents and parents in our clinic regarding their views on transition and their perceived health care needs during transition [14]. We assessed outcomes related to education and daily functioning in patients who had attended our pediatric rheumatology transition clinic, and ascertained satisfaction with the transition process as well as the rate of follow-up in the adult clinic.

## Methods

The Pediatric Rheumatology Transition clinic at the IWK Health Centre, a children’s tertiary hospital in Halifax, Nova Scotia, Canada, began in 1994. It is a dedicated clinic for adolescents with chronic rheumatic diseases in the transition period and is held three to six times per year. The clinic takes place in the pediatric rheumatology clinic and is attended by at least one pediatric rheumatologist, an adult rheumatologist with an interest in youth, a pediatric rheumatology nurse, and a pediatric physiotherapist. Generally, we attempt to transfer patients when they have finished high school and their disease is quiescent, therefore the first transition clinic appointment usually occurs in the 2 years prior to completion of high school. The timing and number of scheduled transition clinic visits before transfer to the adult system is individualized for each patient based on our assessment of need, both from a medical and psychosocial point of view. We do not limit discussions around transition to transition clinics; it is our practice to begin addressing these issues much earlier during regular clinic appointments, however, the transition clinics are dedicated to the older teen group (e.g. younger children are not seen during this clinic) and transition/impending transfer is the primary focus of the health care team. At the first transition clinic visit, the adolescent is seen in consultation by the adult rheumatologist who reviews their case with their pediatric rheumatologist. Both physicians complete the assessment together with the patient, and other members of the pediatric rheumatology team assess the adolescent as needed to discuss transition issues. It is standard practice to see the patient alone, with the parent joining at the end of the visit. During follow up visits, either the adult or pediatric rheumatologist does the primary assessment, followed by both physicians seeing the patient to discuss management and transition plans, and members of the allied health pediatric team addressing transition issues. Depending on disease activity, adolescents may be seen in the pediatric rheumatology clinic for regular care by the pediatric team between transition clinic appointments. The transition clinic serves patients from the three Maritime Provinces. The majority of patients who live locally are transferred to the adult rheumatologist who attends the transition clinic, while most other patients are transitioned to rheumatologists in their region.

In 2003, questionnaires were mailed to 151 patients who had attended or were currently attending the transition clinic. The questionnaire included demographic data, diagnosis, and whether respondents had ongoing adult rheumatologic care. The charts of all the patients transferred to the adult rheumatologist attending the transition clinic were reviewed to assess patient compliance with follow-up. Respondents were asked to rate their overall satisfaction with the transition clinic using a 10 cm visual analog scale (VAS) (anchored at 0 with “completely unsatisfied” and at 10 with “completely satisfied”). They also indicated how well specific items related to the transition process were addressed (anchored at 0 with “not at all” to 10 with “addressed completely”). The items included in the survey included those that would be included in an adolescent health assessment and transition-specific items, specifically those that would impact on independent health behaviors and self-advocacy [4, 15]. To characterize the sample, health status and quality of life were assessed by two well-validated measures, the Medical Outcomes Study 36 item Short Form (SF-36), and the Stanford Health Assessment Questionnaire (HAQ) measured arthritis-specific functional disability [16, 17]. The SF-36 comprises 36 items in 8 domains: physical functioning, role physical, bodily pain, general health perceptions, energy and vitality, social functioning, role emotional, and mental health. Domain scores range from 0 to 100 with higher scores reflecting more normal function. The HAQ consists of 19 items in 9 domains: dressing and grooming, arising, eating, walking, hygiene, reach, grip, activity and sexual function. Scores of 0 to 1 are generally considered to represent mild to moderate difficulty, 1 to 2 moderate to severe disability, and 2 to 3 severe to very severe disability.

The data were collated and descriptive data analysis was performed using Microsoft®Excel ® 2008 for Mac, Version 12.3.6. When analyzing satisfaction and how well items were addressed we elected to define high as > 7, moderate as 5–7, and poor as <5 on the 10 cm VAS scale. SF-36 scores were compared with age-matched norms determining how many scores were more than 1 and 2 SDs below the mean for each domain [16]. The median values of the transition clinic overall satisfaction scores, the perception of disease-control at transfer, HAQ scores and general health perception scores of the SF-36 were compared between those subjects who reported regular follow-up in adult rheumatology care and those who reported no regular follow-up using Stata® v11.1, 2009. This study was approved by the IWK Health Centre Research Ethics Board.

## Results

Fifty-one completed questionnaires were returned giving a response rate of 34 %. Demographic data are presented in Table 1. The median age at the time of questionnaire completion was 22.0 years (range 17 to 27 years). The most common condition was JIA (82 %). The majority of respondents resided in Nova Scotia (82 %). Of the 46 respondents who answered questions on educational level, 16 completed only high school, and of the remaining, 5 completed or were enrolled in community college, 24 completed or were enrolled in a university undergraduate program, and one was enrolled in a university post-graduate program. Fourteen percent of respondents reported being regular smokers (>10 cigarettes per day), 51 % reported alcohol use at least once per month over the past year, and 80 % reported being sexually active.

**Table 1:** Demographic characteristics of subjects

Demographic variable	Result
Age (years), median (range)	22 (17–27)
Gender, M:F	11:40
Ethnicity, n (%)	
Caucasian	49 (96.0)
Asian	1 (2.0)
No response	1 (2.0)
Diagnosis, n (%)	
Juvenile idiopathic arthritis	42 (82.3)
Systemic lupus erythematosus	4 (7.8)
Juvenile dermatomyositis	1 (2.0)
Other	4 (7.8)
Province of residence, n (%)	
Nova Scotia	42 (82.3)
New Brunswick	7 (13.7)
Prince Edward Island	2 (3.9)
Marital status, n (%)	
Single	40 (78.4)
Common-law	6 (11.8)
Married	5 (9.8)
Highest Educational level, n (%)	
Completed High school	16 (31.4)
Community college^a^	5 (9.8)
University undergraduate^a^	24 (47.1)
University postgraduate^a^	1 (2.0)
No response	5 (9.8)
Smoke >10 cigarettes daily, n (%)	7 (13.7)
Use alcohol at least once per month over past year, n (%)	26 (51.0)
Sexually active, n (%)	41 (80.3)

^a^Completed or enrolled

Most patients (88 %) were transitioned between 17 and 20 years of age. Five patients were transferred at the age of 16. Twenty-nine of 50 respondents (58 %) felt they were transitioned at the appropriate age, whereas 6 (12 %) felt they were too young and 3 (6 %) felt they were too old (all of these subjects were transferred between 17 and 20 years of age). Twelve respondents (24 %) felt age did not matter. Of 40 patients who indicated the number of transition clinics they had attended, 17 subjects (43 %) attended a single transition clinic prior to transfer, 18 (45 %) attended 2 to 4, and 5 (12 %) attended five or more. Of 47 patients responding to the question regarding the appropriate number of transition clinics, 26 (55 %) felt they attended the appropriate number of transition clinics. Fourteen of these subjects (54 %) had attended 2 to 4 clinics and 7 (27 %) had attended only a single transition clinic. Of the 9 subjects who felt they attended too few clinics, 7 (78 %) had attended only one clinic. One patient felt they attended too many clinics and 11 respondents felt it didn’t matter how many transition clinics they attended.

Quality of life data, as assessed using the SF-36, are presented in Table 2. There were impairments in all domains, with approximately one quarter of respondents scoring greater than 2 standard deviations below the mean in 5 out of 8 domains, including physical functioning, role physical, bodily pain, social functioning and mental health. The median score on the HAQ disability index was 0.125 representing mild disability (range 0 to 1.5). Twenty-six (51 %) respondents had a HAQ score of zero, representing no or minimal disability while 25 (49 %) had a score greater than zero, representing some degree of disability. Of these, 5 subjects (9.8 %) had a score of 1 to 1.5 indicating moderate disability. All respondents answered questions on how much they had reduced their daily activities because of their health over the previous six months. Twenty-eight respondents (55 %) reported having reduced their activities on an average 27 days over the past 6 months (range 1 day to everyday). Nine respondents (19 %) were completely unable to carry out their activities on an average of 3.8 days (range 1 to 15 days). Seventeen respondents (44 %) had missed work on an average of 3.3 days (range 1 to 7 days) and 13 (33 %) were unable to complete all their work-related activities on an average of 2.7 days (range 1 to 5 days).

**Table 2:** Quality of life (SF-36) of subjects and comparison with normative data for 18 to 25 year olds

SF-36 Domain	Median	Range	>1 SD (%)	>2 SD (%)
Physical functioning	90	17-100	15 (29)	10 (20)
Role physical	100	0-100	19 (37)	14 (27)
Bodily pain	100	0-100	14 (27)	14 (27)
General health perceptions	75	25-100	13 (25)	4 (8)
Energy and vitality	62	10-100	15 (29)	4 (8)
Social functioning	60	0-100	29 (57)	14 (27)
Role emotional	72	12-100	7 (14)	1 (2)
Mental health	62	10-100	20 (39)	14 (27)

> 1 SD is number of patients more than 1 standard deviation below the population mean; > 2 SD is number of patients more than 2 standard deviations below the population mean

Overall satisfaction with the transition clinic and ratings of how well individual items related to the transition process were addressed in the clinic are found in Table 3. The mean satisfaction score with the transition clinic, as measured on a 10 cm VAS, was 7.3 (SD 2.6). Thirty patients (59 %) rated overall satisfaction >7. The mean scores of how well items were addressed ranged from 4.9 to 7.6 out of 10 (mean 6.1). For a number of items, some respondents rated the item as high (>7) and an equal or close to equal number rated the item as poor (<5). One example is the item in which subjects felt involved in decision-making; 45 % rated this in the high satisfaction range (>7) and 29 % rated this in the poor satisfaction range (<5). Four of the 14 specific items assessed were given a rating of >7 by the majority of subjects: learned about side effects of medications (51 %), learned about future living with their disease (53 %), felt confident in disease management (53 %), and disease well-controlled at transfer (53 %). However, even within these items, a proportion of respondents rated the item in the poor range (<5). Five items had at least a third of respondents scoring poorly (<5): smoking adequately addressed (33 %), alcohol use adequately addressed (35 %), drug use adequately addressed (33 %), sexual health adequately addressed (33 %), and learned about new developments related to their disease (45 %). Again, although these 4 items had the highest proportion of low scores, some subjects rated the same item in the high range. Five items appeared to be distributed more evenly: felt well-informed as a patient, learned to manage a disease flare, learned to manage pain, felt involved in decision-making, and learned about joint protection.

**Table 3:** Satisfaction with the transition clinic measured on a 10 cm VAS

Item	Mean Score ± SD (range)	>7 n (%)^a^	≤7and ≥5 n (%)	<5 n (%)
Overall satisfaction with transition clinic	7.3 ± 2.6 (0.1-10)	30 (59)	9 (18)	8 (16)
Smoking was adequately addressed	5.6 ± 3.8 (0–10)	20 (39)	7 (14)	17 (33)
Alcohol use was adequately addressed	6.0 ± 3.6 (0–10)	23 (45)	6 (12)	18 (35)
Drug use was adequately addressed	5.8 ± 4.0 (0–10)	24 (47)	6 (12)	17 (33)
Sexual health was adequately addressed	5.9 ± 3.6 (0–10)	22 (43)	8 (16)	17 (33)
Felt well-informed as a patient	6.5 ± 2.8 (0.8-10)	23 (45)	12 (24)	12 (24)
Learned to manage a disease flare	6.1 ± 3.0 (0.1-10)	20 (39)	13 (26)	14 (28)
Learned to manage pain	6.4 ± 2.7 (0.2-10)	18 (35)	12 (24)	10 (20)
Learned about joint protection	5.7 ± 3.3 (0–10)	16 (31)	11 (22)	14 (28)
Learned about new developments	4.9 ± 3.3 (0–10)	15 (29)	9 (18)	23 (45)
Learned about side effects of medications	6.3 ± 3.2 (0–10)	26 (51)	9 (18)	12 (24)
Learned about future living with their disease	5.9 ± 3.2 (0–10)	27 (53)	4 (8)	16 (31)
Felt involved in decision-making	6.1 ± 3.1 (0–10)	23 (45)	9 (18)	15 (29)
Felt confident in disease management	6.9 ± 3.1 (0.2-10)	27 (53)	8 (16)	11 (22)
Disease was well-controlled at transfer	7.6 ± 2.7 (0.1-10)	34 (67)	6 (12)	6 (12)

^a^Percentages per item do not add up to 100 due to incomplete fields by subjects

Thirty-seven out of fifty (74 %) respondents reported having regular appointments with an adult rheumatologist, whereas the others reported not attending regular appointments. There were no differences between these two groups when comparing the overall transition clinic satisfaction score, the perception of how well disease was controlled at transfer, HAQ score and general health perception score of the SF-36 (Table 4). All of those reporting no regular follow-up had been transferred to adult rheumatologists other than the individual involved with the transition clinic. Eight of these patients had JIA, 2 had SLE, and 3 had “other” rheumatic conditions. When office charts of the adult rheumatologist who participates in the transition clinic were reviewed, 92 % of patients had attended all or most of their follow-up appointments in the adult clinic.

**Table 4:** Comparison of subjects reporting regular follow-up with adult rheumatology care and those reporting no regular follow-up with adult rheumatology care

Variable	Regular follow-up median scores (n = 37)	No regular follow-up median scores (n = 13)	*p*-value*
Overall satisfaction with transition clinic	8.6	6.8	0.13
Disease well-controlled at transfer	8.4	8.3	0.50
HAQ score	0.125	0.125	0.68
SF-36 general health perceptions	57	77	0.12

*Mann–Whitney *U* Test

## Discussion

A number of studies have described various transition processes and explored the transition needs of adolescents with a chronic illness and their families. Most descriptions are confined to specific health conditions within a subspeciality clinic such as our own [18]. There are a number of position statements and guidelines to aid health care providers in delivering the best care to this patient population [6, 8]. We drew upon these resources as well as information obtained from our previous work when we developed our transition clinic [14]. There has been much less research evaluating transition clinics, making the current study an important contribution to the literature.

The majority of respondents in our study were Caucasian females with JIA, which is consistent with the makeup of the clinic population. Of the 90 % responding to questions about education, all had completed high school and 65 % were enrolled in or had completed post-secondary education. Canadian census data from 2006 indicate that 79.5 % of males and 83 % of females in Nova Scotia attain at least a high-school education [19]. Considering the age range of our subjects, our findings are consistent with previous studies suggesting that young adults with JIA fare at least as well as the general population in terms of educational outcomes [20]. A number of subjects demonstrated poorer health-related quality of life when compared with population norms, and half had some degree of disability as demonstrated by the HAQ. The functional outcome of our patient population is comparable with findings reported in a recent review of adult outcomes of patients with JIA, where approximately 40 % of young adults are somewhat limited in their functional capacity and 10 % are in need of assistance to manage daily routines [21].

Most respondents in our study reported overall satisfaction with the transition clinic, with a mean of 7.3 on the VAS. It appears, however, that some individual items are better addressed than others, and in general there is room for improvement, as all items received a proportion of poor ratings (<5 on the VAS). Items related directly to their disease, such as learning about side effects of medications and confidence in disease management, were reported to have been more completely addressed. There are a number of potential reasons for higher satisfaction in these domains. Many patients were diagnosed as young children and would have had many years to become familiar with their disease and treatments prior to the transition period. Secondly, our previous research indicated that one of the most important factors for adolescents in determining readiness for transfer was confidence in managing their disease. This would therefore likely have been a major focus of the health care providers in the clinic. Our findings are similar to those of McDonagh *et al.* in which adolescents and young adults felt that assuming primary responsibility in managing their health-care was an essential part of transition [22]. In addition, it is likely that our practice of seeing patients independently of their parents contributed positively to overall confidence in self-management. The highest scoring item was satisfaction with disease-control at transfer, suggesting that our practice of delaying transfer of patients if there is evidence of uncontrolled disease should be continued whenever possible.

Items in which a third of respondents provided a poor rating of the item being adequately addressed (<5 on the VAS) included disease non-specific items: addressing smoking, alcohol, drugs and sexual health. These poorer scores may be due to lack of discussion around these topics or that these items were addressed in a manner that was not optimal for the patient, or a combination of both. It is important to note that many respondents also rated the same items in the higher range (>7 on the VAS). There are different views regarding the perceived importance and desire to discuss items such as these depending on who is asked. In our previous survey of patients of a similar age, we found that only a third felt that it was important to discuss these issues; similar findings were reported in a study of adolescents with cystic fibrosis [14, 23] Perhaps not surprisingly, however, most parents surveyed in our previous study felt there was a need for education around these topics. The Society for Adolescent Medicine’s position paper on transition states the importance of addressing topics such as sexuality and substance use in transitional health programs [7]. Similarly, as health care providers, we feel that discussion around these issues is critical given the potential toxicities of medications in youth who have a high likelihood of using alcohol and are likely to be sexually active. Developing methods that will effectively address these issues during the transition process is an important area and deserves further appraisal and study.

Another poorly rated item was learning about new developments in their condition, with a mean score of 4.9/10. Our previous work demonstrated this to be the highest perceived educational need of adolescents, highlighting a deficit in our care and the importance of improved knowledge translation initiatives. This need and how best to address it deserves further exploration.

In our clinic, 88 % percent of patients were transferred between 17 and 20 years of age, and most felt this was appropriate. A small number of individuals, all 17–20 years of age, felt they were either too young or too old at transfer. Our approach, which allows individualizing the time of transfer, appears satisfactory to most patients. From a health systems point of view this is noteworthy as it relies on an institution’s policy to allow patients to be followed beyond age 16 if they have a chronic illness. Most respondents felt that they had attended the appropriate number of clinics; in most cases this was between two to four. Our present model is constrained by the number of times the adult rheumatologist is able to attend a combined clinic at the pediatric hospital. Seven of the 9 respondents who reported attending too few transition clinics had attended only one. There may be a role for involving patients in the decision-making process regarding the number of transition clinics to be attended prior to transfer in the future.

An alternative model of transition is to have a young adult clinic that is a stepping-stone between pediatric and adult care. A number of centres in Canada have developed clinics for Young Adults with Rheumatic Diseases (YARD clinics) in which young adults are seen in a clinic specifically for youth aged 18 to 25 [24]. Further work to evaluate the effectiveness of different models of transition-related care will be important in order to inform clinicians and administrators regarding the best and most cost-efficient care-model for this population.

Approximately 25 % of respondents in our study reported no regular follow-up by an adult rheumatologist, comparable to the study by Hersh *et al.* in which 30 % of subjects missed their first scheduled adult rheumatology appointment and the rate of missed appointments in the post-transfer period was 21 % [12]. It is important to note that all of the respondents reporting no follow-up in our study were those transferred to other rheumatologists (i.e. not the adult rheumatologist involved in the transition clinic), whereas almost all patients followed up by the adult rheumatologist in the transition clinic attended all or most of their appointments, as confirmed by chart audit. This suggests that continuity of care may be an important factor in achieving improved adherence to follow-up after transfer.

This study has a number of limitations. The response rate from our study was only 34 %, leading to limited statistical power and potentially leading to selection bias. Secondly, our primary outcomes were satisfaction and rating of the completeness of care. Satisfaction and completeness of care are complex constructs; we were not aware of a validated measure of satisfaction that would apply to our study population, therefore we chose to use a 10 cm VAS to measure these outcomes. While this provides us with useful information regarding perceived gaps in care, there are likely limitations to this method of measurement and importantly our study does not address whether these outcomes correlate with other outcomes such as knowledge acquisition, self-management behaviours or disease outcomes. It also does not provide information regarding how items could be better addressed. Finally, our questionnaire did not address perceptions regarding completeness of addressing psychosocial and mental health concerns in the transition clinic, an area which we feel needs future study.

## Conclusion

This study describes satisfaction with the transition experience of young adult rheumatology patients after transfer to adult care. Most reported overall satisfaction with the transition clinic, and most respondents felt that being transferred between 17 and 20 years of age was appropriate. However, their perception of how adequately various transition issues were addressed was highly variable. Future research should explore patient preferences regarding discussions around substance use and sexual health in particular, and should aim to improve knowledge translation to provide adolescents with information on new developments related to their illness. Our study reports a high follow-up rate in adult care for a subset of patients attending a rheumatology transition clinic. We speculate that this may be in part related to the continuity of care provided by our joint pediatric-adult clinic transition model. Improving the transition process for those patients transferred to adult rheumatologists other than the specific adult rheumatologist attending the clinic should be a priority for future transition work at our institution, as these patients appear to be at higher risk of being lost to follow-up.
